# Isolated primary hydatid cyst of small intestinal mesentery: an exceptional location of hydatid disease

**Published:** 2012-09-23

**Authors:** Mohammed Najih, Ali Chabni, Gilles Attoulou, Rajae Yamoul, Mbarek Yakka, Abdelkader Ehirchiou, Siffedine AlKandry

**Affiliations:** 1 Visceral surgery department, Military hospital Mohammed V, Rabat, Morocco

**Keywords:** Hydatid cyst, small gut mesentery, subtotal cystectomy

## Abstract

Hydatid disease is an endemic problem in some areas of the world. Common sites include liver and lungs. We report an unusual case of isolated primary Hydatid cyst of small gut mesentery. Characteristics of this uncommon location, mechanism, diagnostic difficulties, and treatment are discussed.

## Introduction

Hydatid cyst may involve any organ or any part of the human body. The disease occurred more frequently in liver and lungs [1]. Mesenteric hydatid disease is usually secondary to spontaneous or iatrogenic rupture of liver or splenic cyst. His primitive form is an exceptional condition. We report an unusual case of primary mesenteric hydatid cyst and discuss mechanism, diagnosis difficulties and therapeutic management of this disease.

## Patient and observation

A 43-year-old man presented with a 10 month history of intermittent attacks of abdominal pain, abdominal distention, recurrent vomiting and nausea. There was no past history of surgery and the review of the family was unremarkable. Abdominal examination revealed a palpable mass (8cm x 3cm) at the peri-umbilical region, firm with restricted mobility.

**Figure 1:**
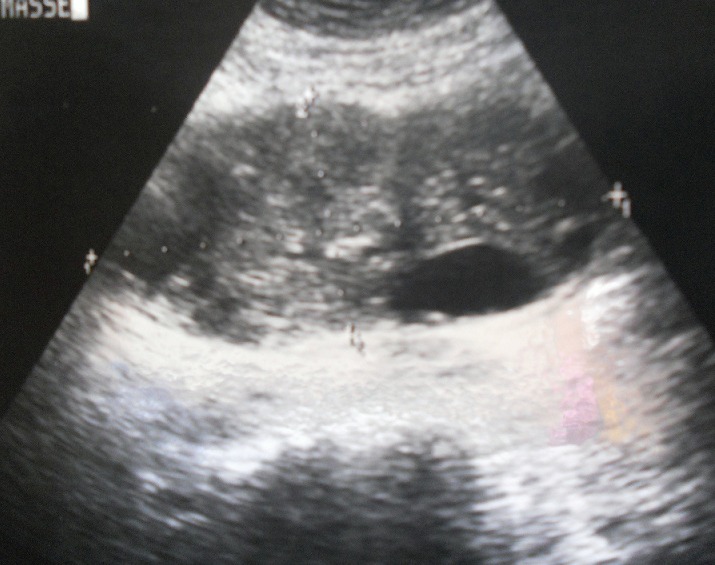
Abdominal ultrasonography (USG) showed a heterogeneous intraperitoneal mass

**Figure 2:**
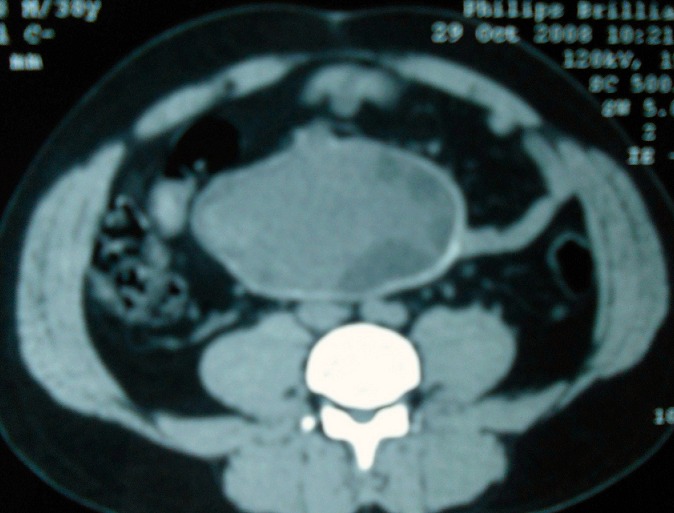
CT scan revealed a large intraperitoneal cyst, with heterogeneous contents

Laboratory investigations were unremarkable. Abdominal ultrasonography (USG) showed a heterogeneous intraperitoneal mass measuring 9 cm x 5cm (Figure 1). Abdominal computed tomography (CT) scan revealed a large intraperitoneal cyst, regular with calcifications in the wall and heterogeneous content; measuring 12 cm x 7 cm; this mass seems getting an intimate report with duodenum; no other lesion was demonstrable in any organ (Figure 2). Hydatid serology was negative. Chest radiography was normal. A provisional diagnosis of duodenal duplication or simple mesenteric cyst was made.

**Figure 3:**
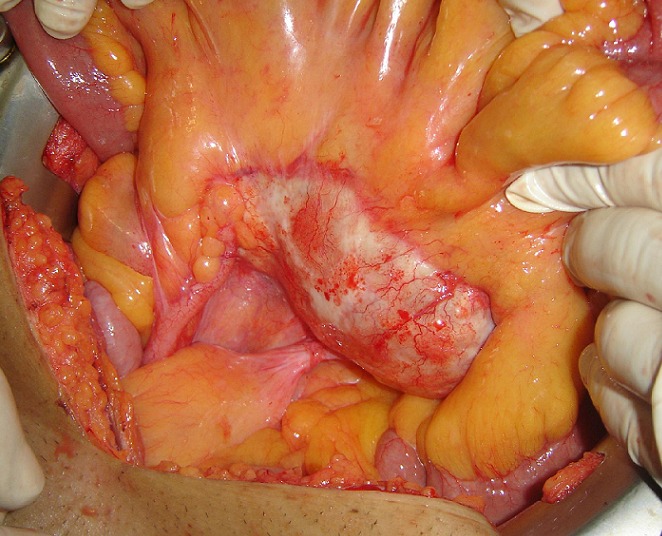
Intra operative photograph: mesenteric cyst

**Figure 4:**
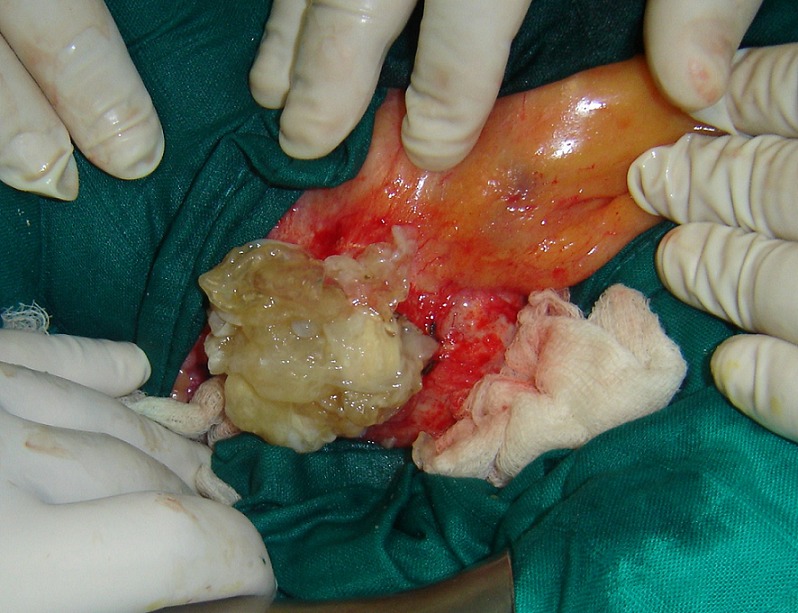
The cystic was opened and hydatid-laminating membrane and daughter cysts were found

Laparotomy showed a cyst of the small ileal mesentery (Figure 3, Figure 4). When the cystic was opened a hydatid-laminating membrane and daughter cysts were found confirming the hydatid cyst. The cyst was irrigated with a scolicide solution (hydrogen peroxide), and a subtotal cystectomy was performed. Rest of the abdomen did not reveal any other cyst and there was no history of any previous hydatid disease in her, making a diagnosis of primary hydatid cyst for sure. The postoperative period was uneventful and the patient was discharged on 8th postoperative day. No recurrence was noted after a decline of 34 months.

## Discussion

The echinococcus or hydatid disease (HD) is an endemic problem in some areas of the world including Mediterranean regions. Liver and lung are the most common organs involved in hydatid disease [1–3]. Small intestinal mesentery hydatid cyst is unusual location, a few cases was reported in the literature. The mechanism of infestation is not clear; dissemination via lymphatic or systemic circulation has been implicated as a possible route [2].

There are no specific symptoms of mesenteric HD and the disease usually remains asymptomatic for years. Clinical manifestation is due to mass effect of enlarging abdominal cyst [4]. For an unusual localization site the diagnosis can be difficult; all abdominal cystic lesions including mesenteric, pancreatic, gastrointestinal duplication, ovarian cysts and lypmangioma, must be considered in the differential diagnosis [3].

The combinations of radiologic and serologic tests especially in patients living in the endemic areas contribute to the diagnosis. USG is the first line of screening for abdominal hydatidosis [5]. CT scan is of particular importance in the designation of surgery strategy [3]. Complement fixation test is positive in approximately 65%, and indirect hemagglutination test and ELISA have approximately 85% sensitivity. The common complications are hydatid peritonitis (due to rupture of the cyst responsible of anaphylactic reaction), infection of the cyst and compression of adjacent organs responsible for an occlusive syndrome [6].

The treatment of choice is principally a careful and complete surgical excision; the partial or subtotal cystectomy can be performed to avoid to adjacent organs injury [7]. The use of hypertonic saline or hydrogen peroxide solutions before opening the cavities tends to kill the daughter cysts and therefore prevent further spread or anaphylactic reaction. Mebendazole or albendazole are used as adjuvant therapy to surgery to prevent recurrence however, in the case the disease recurrence or multiple locations, chemotherapy should be used routinely [3, 7, 8]. In our case we preferred the subtotal cystectomy to keep the intestinal vasculature safe.

## Conclusion

The hydatid disease is very common in North Africa. Mesenteric primary hydatid cyst is an unusual site. In endemic areas HC should be considered in the differential diagnosis of cystic lesions within the abdominal cavity. Surgery is the treatment of choice. But prevention is the best way to reduce the incidence of this disease.

## References

[1] Fazili A, Wani NA, Khan TS, Mir AR (2002). Hydatosis: rare presentations. JK Practioner.

[2] Khare DK, Bansal R, Chaturvedi J, Dhasmana JP, Guta S (2006). Primary peritoneal echinococcosis masquerading as an ovarian cyst. Indian J Surg..

[3] Ghafouri A, Nasiri S, Shojaeifar AF, Mobayen MR, Tahamtan M, Nazari M, Gharib Doust (2010). Isolated primary hydatid disease of omentum; Report of a case and review of the literature. Iran J Med Sci.

[4] Utpal De (2009). Primary abdominal hydatid cyst presenting in emergency as appendicular mass: a case report. World Journal of Emergency Surgery.

[5] Godara R, Dhingra A, Ahuja V, Garg P, Sen J (2007). Primary peritoneal hydatidosis: Clinically mimicking carcinoma of ovary. Internet J Gynecol Obstet.

[6] Astarcioglu H, Koçdor MA, Topalak O, Terzi C, Sokmen S, Ozer E (2001). Isolated mesosigmoidal hydatid cyst as an unusual cause of colonic obstruction: report of a case. Surg Today.

[7] Tajdine MT, Daali M (2007). Kyste hydatique pelvien isolé: à propos de 1 cas pédiatrique. Arch Pediatr.

[8] Badi M, Arifi M, Kaddouri N, Abdelhak M, Benhmamouch N, Barahioui M (2003). Peritoneal hydatidosis in children. Report of a historical case. Arch Pediatr.

